# YAP Signaling in Glia: Pivotal Roles in Neurological Development, Regeneration and Diseases

**DOI:** 10.1007/s12264-024-01308-w

**Published:** 2024-11-06

**Authors:** Lin Lin, Yinfeng Yuan, Zhihui Huang, Yongjie Wang

**Affiliations:** 1 School of Pharmacy, Hangzhou Normal University, Hangzhou, 311121 China; 2 Key Laboratory of Element Class Anti-Cancer Chinese Medicines; Engineering Laboratory of Development and Application of Traditional Chinese Medicines, Collaborative Innovation Center of Traditional Chinese Medicines of Zhejiang Province, Hangzhou Normal University, Hangzhou, 311121 China

**Keywords:** YAP, Hippo pathway, Neuroglia, Nervous system, Nervous system diseases

## Abstract

Yes-associated protein (YAP), the key transcriptional co-factor and downstream effector of the Hippo pathway, has emerged as one of the primary regulators of neural as well as glial cells. It has been detected in various glial cell types, including Schwann cells and olfactory ensheathing cells in the peripheral nervous system, as well as radial glial cells, ependymal cells, Bergmann glia, retinal Müller cells, astrocytes, oligodendrocytes, and microglia in the central nervous system. With the development of neuroscience, understanding the functions of YAP in the physiological or pathological processes of glia is advancing. In this review, we aim to summarize the roles and underlying mechanisms of YAP in glia and glia-related neurological diseases in an integrated perspective.

## Introduction

The Hippo pathway, highly conserved across species, was initially identified in 1995 when the first core component encoded by the *wart (wts)* gene, homologous to human large tumor suppressor 1/2 (LATS1/2), was found in *Drosophila melanogaster* [1]. Thereafter Hippo kinase, the *Drosophila* homologue of the mammalian Ste20-like kinases, was named for its resemblance to a hippopotamus and was shown to stimulate proliferation arrest and apoptosis [2], leading to the emergence of Salvador–Warts–Hippo pathway in *Drosophila* [3]. In 2005, Yorkie (Yki, the YAP/Transcriptional coactivator with PDZ-binding motif (TAZ) ortholog in *Drosophila*) was isolated as a wart-interacting substrate which is essential for tissue growth [4].

**Fig. 1:**
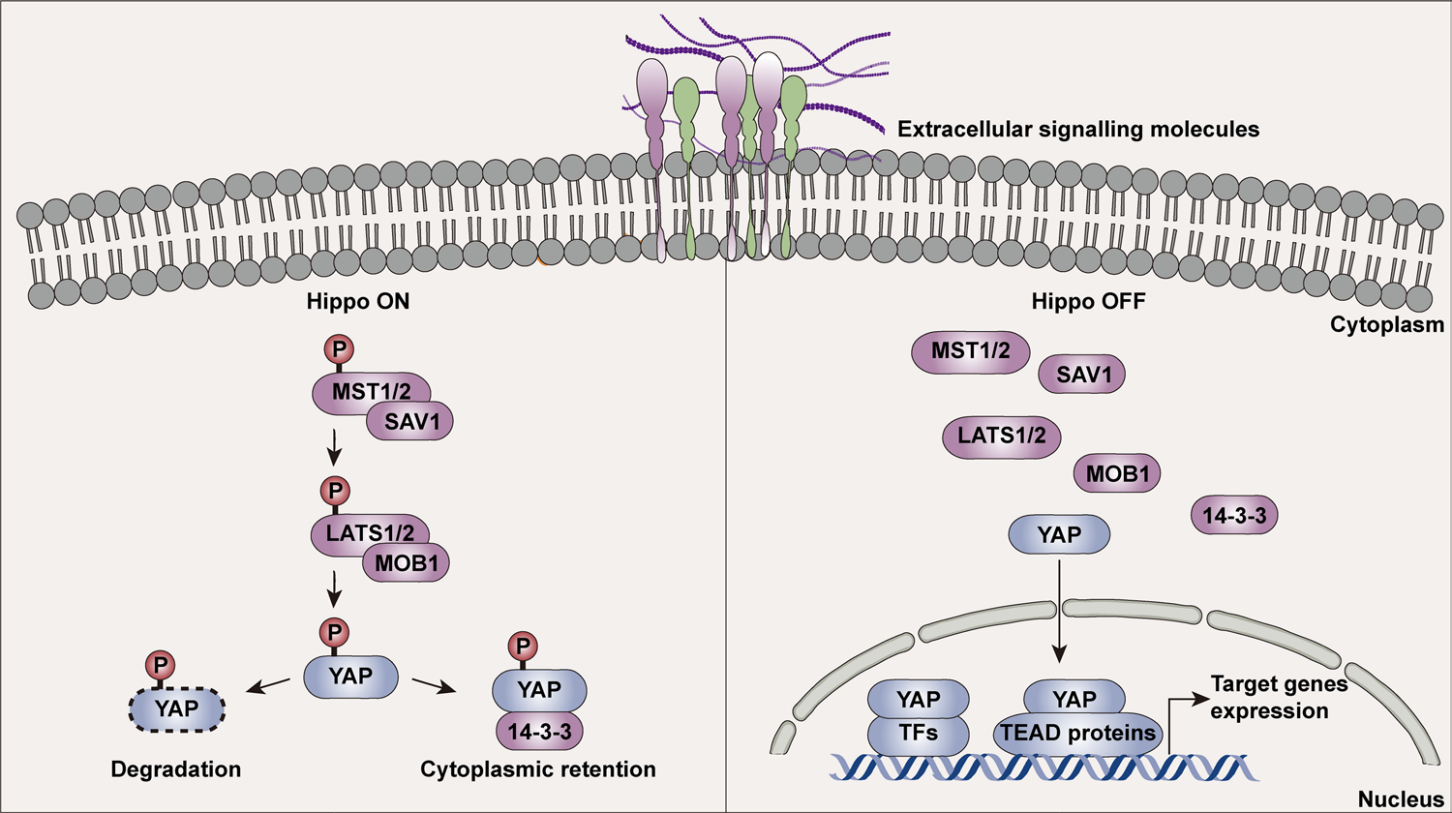
The regulation of YAP by Hippo pathway signaling. Core components of the Hippo pathway can be activated or inactivated by extracellular signaling molecules (such as GPCR, mechanical cues, and cytokines) to control the nuclear-cytoplasmic shuttling of YAP. When the Hippo pathway is active, the core kinases MST1 and MST2 form a complex with SAV1 and phosphorylate the kinases LATS1 and LATS2. YAP therefore is phosphorylated and eventually degraded or retained in the cytoplasm by binding to 14-3-3 protein through direct phosphorylation of MOB1-bound LATS1/LATS2. Conversely, when the Hippo pathway is inactive, YAP is dephosphorylated and shuttles to the nucleus, where it binds with TEAD or other transcription factors to alter gene transcription. LATS, large tumor suppressor; MOB1, mps one binder 1; MST, mammalian STE20-like protein kinase; P, phosphorylation; SAV1, Salvador homologue 1; TEAD, transcriptionally-enhanced associated domain; TFs, transcription factors; YAP, yes-associated protein.

In mammals, activation of the Hippo pathway leads to the phosphorylation of YAP with subsequent cytosolic retention or proteasomal degradation by the upstream MST1/2-scaffolding protein Salvador homologue 1 (SAV1) complex and LATS1/2 kinases-Mps One binder 1 (MOB1) complex (Fig. 1) [5]. Conversely, upon the inhibition of the Hippo pathway, the dephosphorylation and nuclear translocation of YAP are enhanced where YAP acts as a co-factor and requires DNA-binding partners, mostly TEA-domain (TEAD) family members, to associate with cognate cis-regulatory elements on chromatin [6, 7]. Besides, independent of the Hippo pathway, following activation by mechanical cues including extracellular matrix (ECM) stiffness and cell spreading, YAP works as a nuclear relay during mechanotransduction to translate these stimuli into biochemical signals to control cell behaviors [8, 9]. Cells respond to these mechanical cues by modifying their tensional state through integrin adhesion complexes [10]. The actomyosin tension increases with the reconstruction of the cell cytoskeleton and transduces physical inputs to modulate nucleocytoplasmic YAP shuttling [11]. This process requires Rho GTPases, myosin motors, RhoA/rho-associated coiled-coil-containing protein kinase (ROCK), and focal adhesion kinase (FAK) [12, 13].

These signaling pathways affect YAP phosphorylation and activity to alter cell fate determination [14], proliferation [15], senescence [16], tissue regeneration, and organ growth [7]. In neural development, YAP binds to TEAD to enhance the self-renewal and proliferation of neural stem cells [17]. ECM-driven YAP overexpression represses the neurogenesis of neural stem cells with β-catenin to trigger the transcription of neurogenesis effectors like *NeuroD1* [18, 19]. Throughout the expansion of the neural progenitor population, YAP-TEAD signaling induces gene expression, upregulating *cyclin D1* and downregulating *NeuroM* to strengthen proliferation and suppress differentiation [20]. These findings underscore that YAP may be one of the primary regulators of neurons as well as glial cells. Indeed, YAP contributes to glial biology, including proliferation [21, 22], differentiation [23, 24], activation [25], senescence [16, 26], and myelination [27] under both physiological and pathological circumstances in both the peripheral nervous system (PNS) and central nervous system (CNS). Therefore, we provide a synopsis of how YAP engages in glial regulation processes in the nervous system and nervous system diseases, with a discussion of the possibility of further application for therapy in pathological conditions.

## YAP in the Peripheral Nervous System

### YAP in Developing Schwann Cells (SCs)

The PNS contains diverse glial cells, each of which communicates intimately with neurons in heterogeneous positions or of specific types [28]. Among these, SCs are a large population of glia best known for ensheathing axons, producing myelin, and allowing the rapid conduction of nerve impulses. Besides being passive support cells, functioning in a wide range of processes including neural development [29], post-injury nerve repair [30], and tumor growth [31] places SCs in a unique position within the PNS.

#### Schwann Cell Development and Myelination

The SC developmental program begins with neural crest induction from the ectoderm and goes through multiple stages including SC precursors, immature SCs, and pro-myelinating SCs to mature myelinating or non-myelinating SCs. The SC lineage progression suggests the existence of a complex integrated system. Between embryonic day 12 (E12) and the early postnatal period, the immature cells undergo proliferation and expansion. In this phase, neuregulin 1 (NRG1) from axons, laminins from the basal lamina, Notch, and the Hippo pathways are known to drive SC proliferation [32–34]. After that, radial sorting is enabled, during which immature SCs establish a 1:1 ratio with larger diameter axons (~>1 µm) or contact small caliber axons to proceed towards the pro-myelinating phenotype or non-myelinating phenotype, respectively [35]. This process largely depends on the interaction of SCs with the basal lamina containing laminins, collagen, and their corresponding receptors [36]. The downstream intracellular molecules like integrin-linked kinase, Jun activation domain-binding protein 1, and several downstream pathways that regulate the actin cytoskeleton including FAK and Rho GTPases, are also recognized as crucial factors [37–40].

**Fig. 2:**
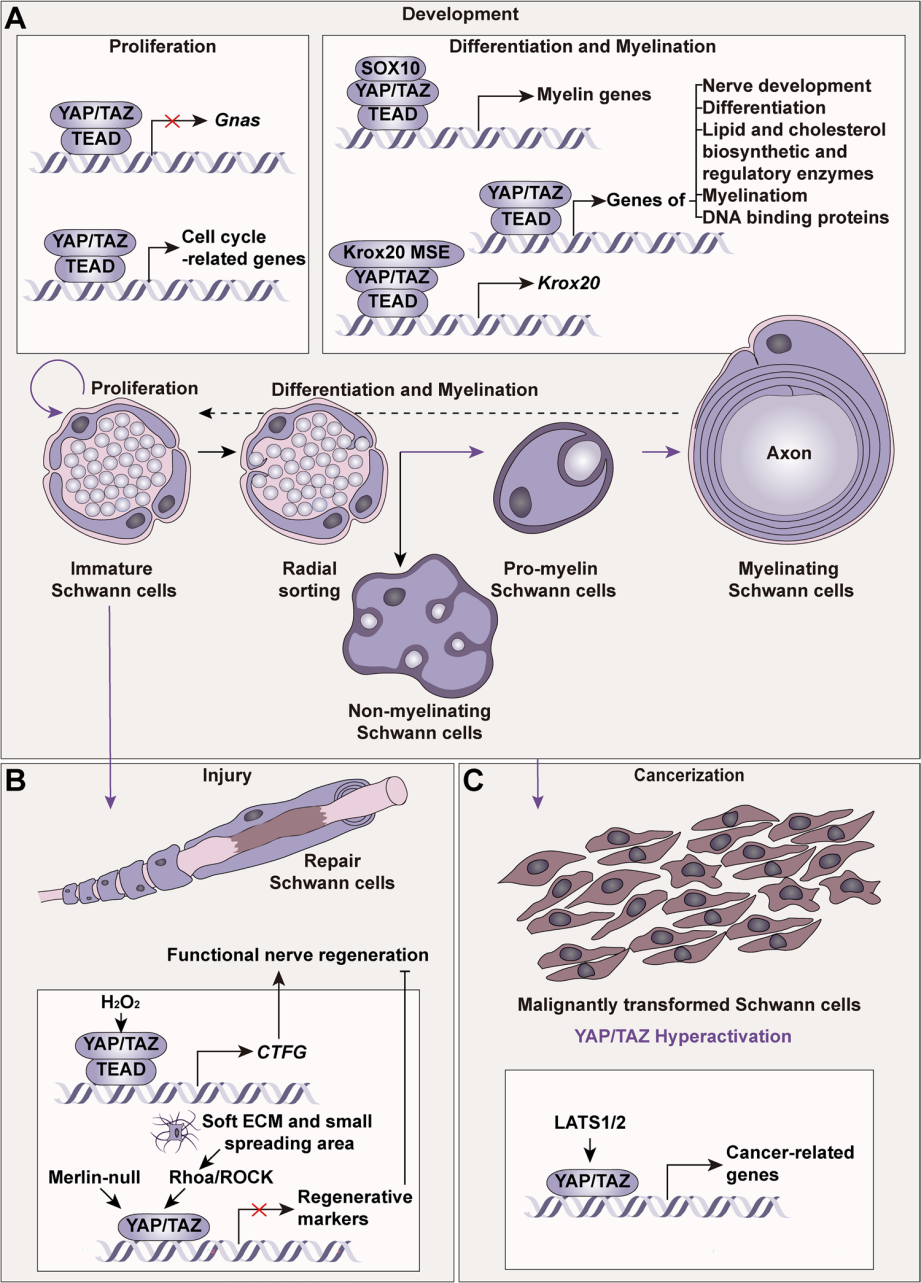
The roles of YAP in developmental stages, repair after injury, and tumorigenesis of Schwann cells. **A** YAP/TAZ activity coordinates proliferation, differentiation, and myelination during SC development (purple arrow), among which the nuclear import of YAP/TAZ induces gene expression in terms of cell cycle-related genes, nerve development, differentiation, lipid and cholesterol biosynthetic and regulatory enzymes, and myelination. **B** YAP/TAZ is essential for the repair of SC proliferation and re-differentiation into myelinating SCs following crush injury. In neuromuscular junction remodeling, H_2_O_2_ upregulates YAP-dependent CTGF expression to facilitate the recovery of neuromuscular function. However, Merlin-null nerves and mechanical cues like well-spread cells and a stiff ECM activate YAP to inhibit SC regeneration. **C** YAP/TAZ hyperactivation can trigger malignant transformation and excessive proliferation of SCs. CNTF, ciliary neurotrophic factor; ECM, extracellular matrix; EMF, electromagnetic field; Krox20, also known as early growth response 2, EGR2; LATS, large tumor suppressor; P0, Protein zero; PMP22, peripheral myelin protein 22; ROCK, Rho-associated coiled-coil-containing protein kinase; TAZ, transcriptional coactivator with PDZ-binding motif; TEAD, transcriptionally-enhanced associated domain.

As the differentiation programs occur, SCs wrap around axons and eventually form compact myelin, in which SCs start to change protein expression including proteins in myelin, such as Protein zero (P0), myelin basic protein, peripheral myelin protein 22, connexin-32, myelin-associated glycoprotein (MAG), and enzymes for the production of cholesterol and sphingomyelin by the expression of transcription factors [41, 42]. During this phase, properly targeted molecular regulation allows opportune number of SCs for differentiation and myelination. NRG1 type III binds to the ErbB2/3 receptor on SCs to promote their proliferation and myelination [43], while the transforming growth factor (TGF)-β signaling pathway is the only signaling pathway to promote SC apoptosis during development so far [44]. Early growth response 2 (EGR2, also known as KROX20), NGFI-A-binding proteins, octamer binding transcription factor 6, and brain 2 class III POU-domain protein contribute to myelination, while c-Jun, paired box gene 3, and sex-determining region Y-box 2 inhibit it. Interestingly, YAP is expressed and largely distributed in the nucleus of SCs during the above phases [15] (Fig. 2A).

Mice bearing loxP-flanked *Yap1* were crossed with P0-Cre conditional knock-out (YAP^P0^ CKO) mice to investigate the role of YAP in myelination. Notably, at postnatal day 20 (P20), when plenty of myelinated axons were present in control mice, the nerves of YAP^P0^ CKO mice showed no abnormalities of radial sorting or myelination. This may be due to the upregulation of TAZ, which interacts and plays abundant roles with YAP [15, 45]. Researchers, therefore, took advantage of different SC-specific YAP/TAZ CKO mice to characterize the contribution of glial YAP/TAZ. The absence of YAP/TAZ in SCs by P0-Cre or desert-hedgehog (DHH)-Cre with the Yap-floxed allele and Taz-floxed allele results in an absence of peripheral myelination, as these SCs fail to invest large-caliber axons at the proper time but delay this to postnatal day 60. The absence further elicits a severely impaired neuromuscular phenotype and near paralysis of the hind limbs [15, 22]. Grove *et al.* [27] deduced that the deficiency of YAP/TAZ by P0-Cre in immature SCs represses their entry into the S-phase to proliferate. Deng *et al.* [22] used proteolipid protein 1 (PLP)-Cre^ERT^ and tamoxifen treatment from postnatal day 0 to postnatal day 9 to exclude the impact of development before birth. This inducible lack of YAP/TAZ in SCs drives severe tremors, ataxia, and eventual lethality, which is a phenotype similar to YAP/TAZ^P0^ CKO mice. This consequence implies that the inability to differentiate into myelinating SCs may primarily cause dysmyelination [46]. However, the current results mainly focus on YAP/TAZ CKO mice. The roles of the nuclear location of YAP require methods to control TAZ expression and further studies.

#### Myelin Elongation

During development, myelin diameter and length increase in coordination and are proportional to the axon diameter. Notably, internodal length is crucial for nerve conduction velocity and closely correlates with nerve length [47]. Interestingly, the expression of a dominant active YAP variant (S127A) significantly increases internodal length and myelin sheath length, while having no impact on myelin thickness [48]. Similarly, loss of YAP/TAZ in PLP-Cre^ERT^ SCs doesn't change myelin sheath thickness and integrity [22]. While NRG1 can upregulate YAP with a slight promotion of internodal length, the polarity protein Crb3 negatively orchestrates YAP through activation of the Hippo pathway to dominate myelin elongation [48, 49].

#### Nucleocytoplasmic Shuttling

The nuclear exclusion of YAP, where YAP shifts to the cytoplasm concomitant with the differentiation of developing cells, has long been considered to be imperative for homeostasis [7]. Since YAP has been shown to be largely located in the nucleus in proliferating, differentiating, and mature myelinating SCs, what controls the activation or inactivation of YAP? In *in vitro* studies, ascorbic acid has been applied with rat SCs plated on dorsal root ganglia (DRG) neurons to initiate myelination. Nuclear YAP/TAZ are detected seven days after ascorbic acid treatment during which proliferation and myelination occur. Another element for consideration is the density of SCs. Deng *et al.* [22] reported that YAP/TAZ is localized to the nucleus in SOX10 (a protein that is present at all developmental stages [50]) ^+^S100β^+^ rat SCs, regardless of high and low density. However, Poitelon *et al.* [15] reported that only a sparse density can initiate nuclear translocation but not a dense or sparse density with blebbistatin, an inhibitor of non-muscle myosin. Likewise, YAP/TAZ retention in the nucleus can also be triggered on glass surfaces with extremely high hardness (4GPa) or in laminin 211 with mechanical stretch. Rather, SCs plated on 0.5 kPa and 40 kPa polyacrylamide hydrogels or 4 MPa polydimethylsiloxane exhibit nuclear exclusion of YAP. Exposure to a 50-Hz electromagnetic field downregulates Crumbs homologues/angiomotin-like-2 protein signaling and the tumor suppressor merlin, thereby decreasing the nuclear localization of YAP in SCs [51]. *Gnas*, encoding Gas-protein, which restrains TAZ/YAP expression, is a strikingly direct transcriptional target of TAZ. This Gas-mediated antagonistic effect induces a YAP/TAZ and Gas-signaling feedback loop which delicately coordinates the balance between SC proliferation and differentiation [22].

#### Downstream Target Genes

Mechanistically, over-activating YAP by dominant active YAP S127A alters myelin genes by changing myelin-related proteins, for example, by increasing EGR2 and MAG and decreasing myelin protein zero [48]. Genome-wide transcriptomic profiling was applied to developing peripheral nerves in P0-Cre; *Taz* cKO-*Yap* cHet mice, in which YAP and TAZ are essential for controlling nerve development, revealed by their regulatory roles in processes such as protein kinase C (*Prkcq*) and adenylate cyclase (*Adcy1*), radial sorting (*Dag1* and *Itga6*), differentiation (*Mag*, *Pmp22*, *Mbp*, *Sox2*, and *Pou3f1*), lipid and cholesterol biosynthetic and regulatory enzymes (sterol regulatory-element-binding protein 2 (*Srebf2*) and *Srebf* target genes [15]. Sophie *et al.* [52] further analyzed this RNA-seq data and identified two DNA binding proteins, Coiled-coil and C2 domain-containing 1B protein and Purine-rich element-binding protein B, which regulate myelination but not SC proliferation.

Subsequent studies have intimated that YAP/TAZ may regulate myelination more directly by the upregulation of critical myelination regulators (e.g., *Krox20* and *Zeb2/Sip1*), myelin genes (e.g., *Erbb2/3*) and cell cycle-related genes (e.g., *Ccnj*, *Pim3*, *Cdk6*, and *Mycn*), and downregulation of myelination inhibitors (e.g., *Hes1* and *Egr1*) [14, 15, 27, 48, 53], thereby mediating developmental myelination. Moreover, the mechanistic target of the rapamycin complex 1 (mTORC1) pathway has emerged as a promising downstream target of YAP to be activated to promote myelination [54, 55]. However, as a transcriptional co-factor, YAP does not act alone and its functional interactions with TEAD transcription factors or others to orchestrate this process are complex and delicate. So, multiple studies are required to fully understand the elaborate roles of YAP in gene transcription or other novel aspects in developing SCs.

### YAP in Mature Schwann Cells and Pathological Conditions

#### Repair and Functional Nerve Regeneration

YAP/TAZ continues to be expressed strongly in SC nuclei until P60, implicating that they also cooperate with fully mature SCs [27] (Fig. 2B). However, injection of tamoxifen from P40 to P100 in PLP1-Cre^ER^ or SOX10-Cre^ER^ with YAP^fl/+^ and TAZ^fl/fl^ showed a phenotype analogous to untreated mice, unraveling a dispensable role of YAP/TAZ in myelin maintenance [23].

In regenerating nerve, where SCs proliferate and reprogram to a repair phenotype, YAP/TAZ expression in SCs dramatically disappears when they lose axon contacts and then reappears after regaining the contacts to facilitate remyelination in a spatiotemporally axon-dependent way. This implies that YAP may function as an inhibitor of axon regeneration, which differs from that in SCs during development [14]. By precise application of ECM stiffness and cell spreading, Xu *et al.* [56] confirmed that soft ECM with a small spreading area activates ROCK/YAP/TAZ to downregulate markers of SC regeneration. Another study suggests that YAP-null in SCs restores the failed regeneration and remyelination caused by injury in merlin-null nerves [57]. Dose- and context-dependent inhibition of axon regeneration *via* YAP in SCs also occurs in inducible YAP/TAZ knockout mice by PLP1-Cre^ERT2^ [14]. When axons reemerge, SCs re-differentiate to myelin-forming SCs to fulfill motor and sensory functions [58]. Grove *et al.* [14] discovered that YAP/TAZ is not required for SC proliferation or transformation into a repair phenotype after injury but is imperative for the remyelination of regenerated axons. Jeanette *et al.* [23] pointed out that SC proliferation and reprogramming are impaired in YAP^fl/+^; TAZ^fl/fl^; PLP1-Cre^ER^ and YAP^fl/+^; TAZ^fl/fl^; SOX10-Cre^ER^ mice and then manifest as c-JUN dysregulation, a lower injury-elicited proliferation rate, and a reduced differentiation capacity of repair SCs. However, at the neuromuscular junction, the local H_2_O_2_ raises YAP/TAZ-mediated connective tissue growth factor (CTGF), a well-defined target of the YAP-TEAD complex. The upregulation of CTGF strengthens SC migration, culminating in the recovery of neurotransmission and polarized axonal elongation [59].

#### Tumorigenesis

YAP/TAZ is not normally involved in mature SC proliferation [14, 23], whereas abnormally high levels of YAP have been shown to elicit excessive proliferation of SCs after nerve injury [57] (Fig. 2C). YAP/TAZ hyperactivation induced by LATS1/2 inactivation sculpts reprogramming to malignantly transformed SCs and their proliferation, subsequent upregulating cancer-related genes [31, 60]. Surprisingly, only concurrent inhibition of YAP and TAZ can block schwannoma cell proliferation [61]. Nonetheless, a recent study demonstrated the effects of YAP on the direct transcriptional regulation of miRNAs in actively proliferating human SC models (hSC2λ) *via* miR-30a. Loss of miR-30a decreases the cell growth rate and increases the apoptosis in SCs along with downregulation of the tumor suppressor protein tyrosine phosphatase non-receptor type 1 (PTPN13) and Kruppel-like factor 9 (KLF9) [62]. Against this backdrop, how appropriate levels of YAP/TAZ are maintained needs to be further clarified.

### YAP in Olfactory Ensheathing Cells (OECs)

**Fig. 3:**
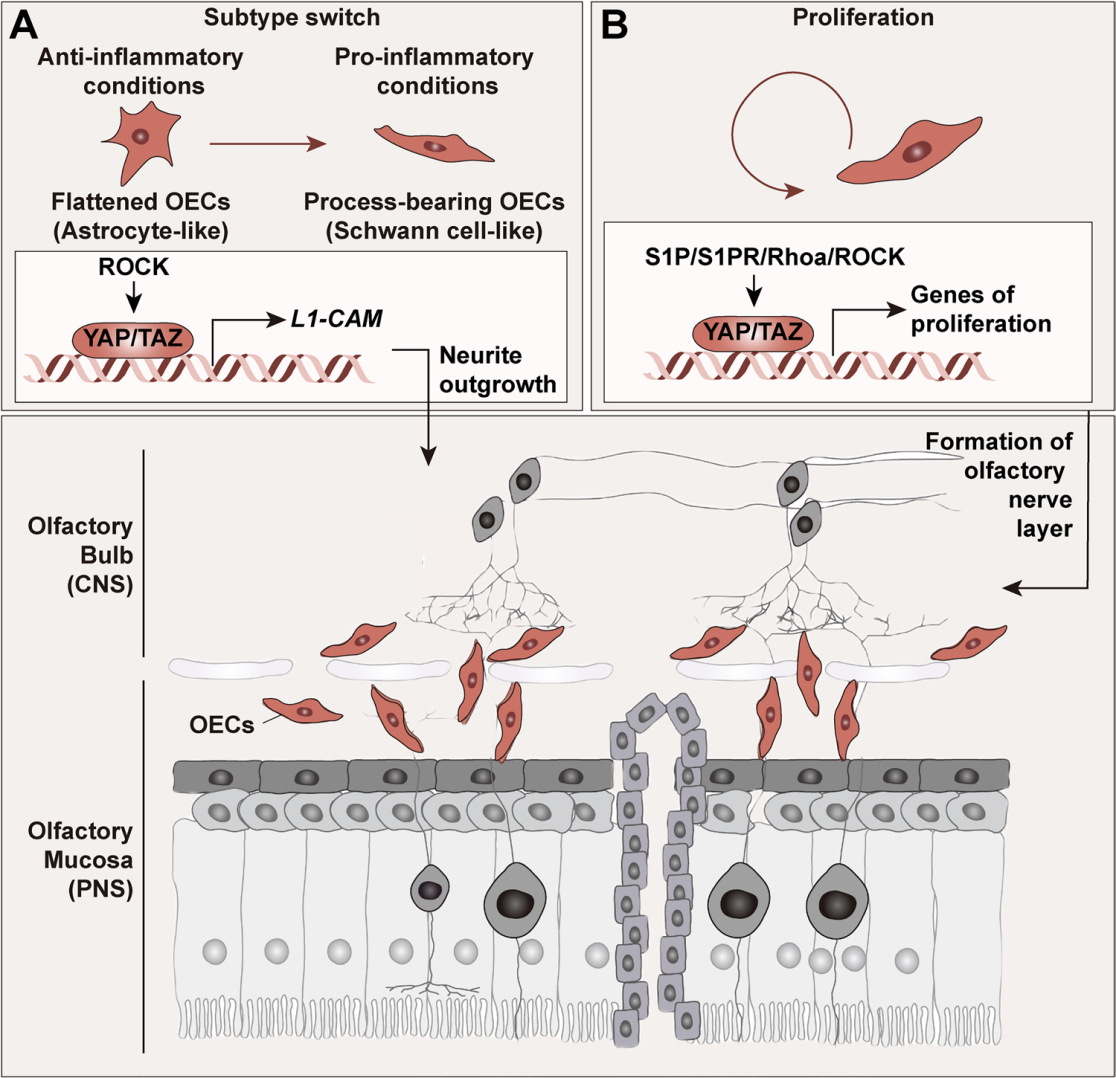
The roles of YAP in olfactory ensheathing cells. OECs are characterized by axonal growth-promoting activity in the olfactory nerve. YAP/TAZ in OECs are upregulated by ROCK and participate in the regulation of proliferation (**A**) *via* the S1P/S1PR/Rhoa/ROCK pathway and switching from an astrocyte-like to a Schwann cell-like subtype of OECs (**B**) (brown arrow), resulting in higher metabolism and migration. These alterations significantly promote the formation of the olfactory nerve layer and neurite outgrowth, which may facilitate OEC transplantation for CNS injuries, as they may improve the regenerative potential and integration of transplanted cells within the injured CNS. CNS, central nervous system; L1-CAM, L1-cell adhesion molecule; ROCK, Rho-associated coiled-coil-containing protein kinase; S1P, sphingosine 1-phosphate; S1PR, S1P receptor; OECs, olfactory ensheathing cells; PNS, peripheral nervous system; TAZ, transcriptional coactivator with PDZ-binding motif.

OECs are glia in the olfactory system that reside in the olfactory epithelium and olfactory bulb, covering the entire olfactory nerve. Their heterogeneous subtypes, classified based on anatomical location, function, and antigenic profile, are thought to be a potential candidate for stimulating regeneration in CNS injuries and demyelinating diseases [63, 64]. The subtypes can switch among each other by alterations of the ECM and cAMP (cyclic adenosine monophosphate, an intracellular factor) [65]. Notably, the ROCK inhibitor Y27632 upregulates neural L1-cell adhesion molecule (L1-CAM) in a YAP-dependent manner, inducing an integrin-focal adhesion signaling-related morphological shift from astrocyte-like OECs to SC-like OECs and enhanced neurite outgrowth [66] (Fig. 3A). Likewise, our evidence showed that the sphingolipid metabolite sphingosine 1-phosphate (S1P) promotes the formation of the olfactory nerve layer by enhancing the proliferation of OECs through the S1P receptor 1 (S1PR1)/RhoA/ROCK/YAP axis, which may also contribute to the potential application of OEC transplantation in treating CNS injuries [67] (Fig. 3B).

## YAP in the Central Nervous System

### YAP in Radial Glia Cells (RGCs)

Glia, comprising roughly half of the cells in the CNS, play a crucial role in coordinating basically all aspects of brain development and function. Among them, RGCs and neuroepithelial cells, the apical neural progenitor cells, are the cells that generate neurons and glia in the developing mammalian cerebral cortex.

#### Cortical Development

**Fig. 4:**
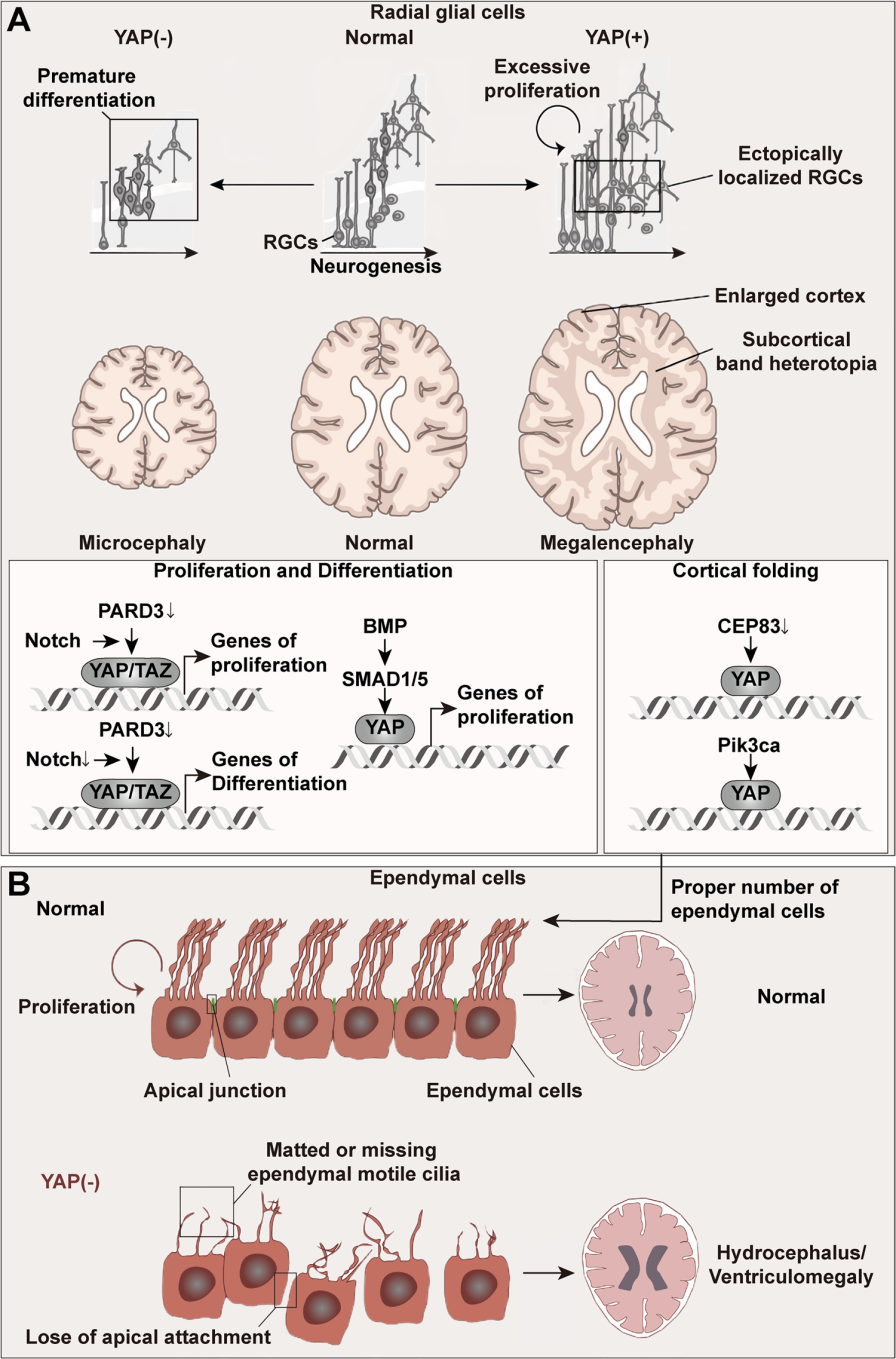
The roles of YAP in radial glial cells and ependymal cells. **A** YAP maintains proper cortical development by orchestrating processes in RGCs. The abnormal expression of YAP in RGCs disrupts the pinpoint processes of RGC proliferation, neurogenesis, localization, and cortical folding, thereby resulting in malformations of the neocortex. Nuclear YAP in RGCs can be upregulated by the coordination of Notch and PARD3 signaling, depletion of CEP83 and Pi3kca. Excessive nuclear YAP localization induces an enlarged cortical volume with subcortical band heterotopia and excessive superficial layer neurons, eventually generating megalencephaly. In comparison, the inactivation of YAP by downregulating the expression of the BMP-SMAD1/5 pathway results in microcephaly with increased early-born neurons, decreased late-born neurons, and fewer RGCs. Moreover, the proper expression of YAP in RGCs maintains a constant number of ependymal cells, while abnormally high or low expression causes hydrocephalus. **B** The disordered YAP in ependymal cells generates fewer ependymal cells, matted or missing ependymal motile cilia, and loss of apical attachment, leading to dysfunction of motile cilia and hydrocephalus. BMP, bone morphogenetic protein; CEP83, centrosomal protein 83; Pi3kca, the catalytic subunit of the PI3K enzyme; PARD3, partitioning-defective 3; RGCs, radial glial cells.

During corticogenesis, glial cells are organized to form the ventricular zone (VZ) to comprise the mammalian cerebral cortex [68]. The RGCs connecting both the ventricle and the basal lamina actively divide and expand to amplify the progenitor pool. After that, they mostly transfer to asymmetric neurogenic division to produce neurons either directly or indirectly through transient amplifying progenitors (e.g., intermediate progenitors). To generate a neocortex with proper function, the size and cellular composition, number, and diversity of neurons must be in the appropriate range, thereby demanding an exquisite balance between the proliferation and differentiation of RGCs [69]. This transition can be impacted in a YAP/TAZ-dependent manner (Fig. 4A). The empty spiracles homeobox gene 1 (EMX1)-Cre; PARD3 CKO mouse-induced deficiency of Partitioning-defective 3 (PARD3, a protein massively expressing PARD6 and atypical protein kinase C (aPKC) to form the PARD complex during development) in RGCs elicits abnormal proliferation and differentiation of RGCs beyond the VZ, resulting in an enlarged cortical volume with subcortical band heterotopia, excessive superficial layer neurons, and seizure susceptibility. However, the triple deletion of EMX1-Cre, PARD3, YAP, and TAZ suppresses the heterotopic formation and symptoms caused in EMX1-Cre; PARD3 CKO mice. During neurogenesis, PARD3 controls YAP/TAZ expression to orchestrate RGC behaviors *via* the regulation of Notch activity. When Notch signaling activity is relatively high, deficiency of PARD3 elevates YAP expression to strengthen symmetrical proliferation. Conversely, at the later stage, the absence of PARD3 leads to low NOTCH and YAP activity, which subsequently promotes differentiation [70]. The activity of the bone morphogenetic protein (BMP) transcription factors SMAD1/5 supports self-amplifying RGC division through the positive regulation of YAP in both chicks and mice. Nonetheless, in Nestin-Cre; SMAD1; and SMAD5 CKO mice, early-born neurons are increased, while late-born neurons are decreased with fewer RGCs owing to a premature switch to neurogenesis, ultimately generating microcephaly [71]. Studies by Lavado *et al.* [72] imply that this premature switch may be attributed to a YAP/TAZ inactivation-induced reduction of cell-cycle speed. Compared to control mice, the top downregulated gene sets in RGCs of EMX1-Cre; YAP; and TAZ CKO brains are Hippo signaling, motile cilium assembly, and cell-substratee junction organization. In accordance with these gene expression patterns, EMX1-Cre; YAP; and TAZ CKO mice show an impaired capacity for protecting ependymal cells and neuroepithelium and a state called hydrocephaly.

#### Cortical Folding

Another characteristic feature of the human brain related to apical progenitor proliferation is the external folded appearance, along with the evolution of human cognition [73]. This cortical folding is critically dependent on basal progenitor cells in the outer subventricular zone, particularly basal radial glial cells [74]. Mice with activating mutations of Pik3ca, the catalytic subunit of the phosphoinositide 3-kinase (PI3K) enzyme, can cause brain overgrowth syndromes comprising megalencephaly, epilepsy, and developmental hydrocephalus. In this model, over-activation of PI3K induces ectopic nuclear YAP^+^ cells in the VZ, apical cell adhesion, and the subsequent focally concentrated progenitor cells and the impairment of Nestin^+^/GFAP^+^ radial glial scaffold fibers leading to dysplastic gyrification and ventriculomegaly [75]. Meanwhile, the absence of YAP/TAZ in Nestin-Cre; YAP; TAZ CKO mice also shows an aberrant shape and distribution of RGCs in the radial glia scaffold during cerebellar development [76].

The centrosome functions as the cell’s microtubule organizing center and the basal body for ciliogenesis in vertebrates. The microtubule organizing activity of centrosomes is indispensable for newly-born neurons to migrate from their birthplace at the VZ [77]. Different from the centrosome located next to the nucleus in other cells, the centrosome in RGCs is positioned far from the nucleus in the apical endfoot at the surface of the VZ [78]. Selective deletion of centrosomal protein 83 (CEP83), which is situated in the centrosomes of RGCs, eliminates the disorganization of microtubules and the associated stretching and stiffening of the apical membrane, thereby activating YAP. The over-activation of YAP causes cortical enlargement and folding, implicating a link between CEP83/YAP signaling, centrosomal abnormalities, and brain overgrowth [79].

Given that both the excessively high and low expression of YAP in RGCs or the VZ is detrimental to brain development, it is important to find out how to constrain the expression of YAP to a suitable range.

### YAP in Ependymal Cells

Within the CNS, ependymal cells form a continuous sheet of ciliated epithelial cells to shape the blood-cerebrospinal fluid (CSF) barrier and the CSF-brain barrier, which separate the CSF from the brain parenchyma [80]. Hence, normal generation, maturation, and integrity of ependymal cells are essential for sustaining CSF flow. The failure of these processes corresponds intimately to hydrocephalus due to cilia defects inclusive of fetal non-communicating hydrocephalus and posthemorrhagic hydrocephalus of prematurity [81, 82] (Fig. 4B). Embryonic Pik3ca activation disrupts YAP-driven development from radial glial cells toward ependymal cells and aggravates hydrocephalus, which can be partially restored by the nuclear YAP inhibitor verteporfin [75]. Similarly, Robinson *et al.* [83] reported a strategy of erythropoietin plus melatonin to remedy posthemorrhagic hydrocephalus of prematurity in rats. The treatment is able to prevent matted or missing ependymal motile cilia, macrocephaly, ventriculomegaly, and cliff aversion *via* normalizing ependymal *Yap* mRNA levels. Park *et al.* [81] found that lysophosphatidic acid deregulates YAP expression and disrupts the localization of YAP at the apical junction in the developing aqueduct, which is vital for the proliferation of ependymal cells and the integrity of the apical lining of the aqueduct, thereby inducing aqueductal stenosis and fetal hemorrhagic hydrocephalus.

### YAP in Retinal Müller Cells (RMCs)

RMCs are the major macroglia in the retina that contact virtually every neuron [84]. Under physiological conditions, RMCs function in the homeostatic and metabolic support of retinal neurons and mechanical tissue, light guidance, regulation of neuronal activity by gliotransmitters, and the release of glutamate and D-serine [85].

#### Retinal Development

**Fig. 5:**
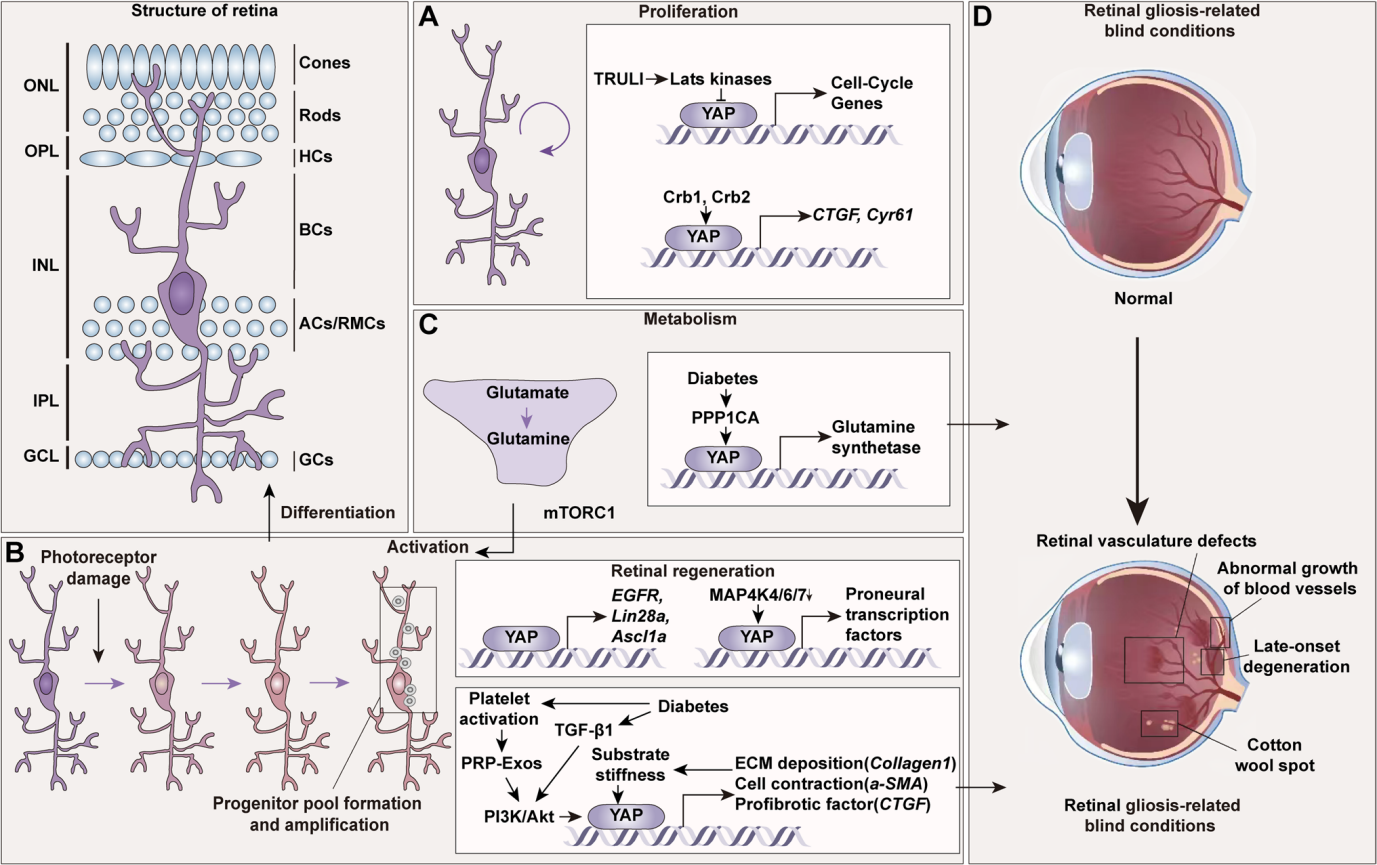
The roles of YAP in retinal Müller cells. YAP in RMCs works under developmental, pathological conditions of the retina (purple arrow). YAP maintains proper development and regeneration through fine-tuned proliferation, activation, and reprogramming, whereas hyperactivation of YAP induced by diabetes or other illnesses tends to aggravate lesions. **A** YAP can promote the proliferation of RMCs through Crb1, Crb2, and the inhibition of Lats kinases. In the postnatal retina, downregulation of YAP leads to abnormal visual responses. **B** The YAP/lin28a/ascl1a and YAP/EGFR axes support RMC proliferation and progenitor cell amplification to promote retinal regeneration in zebrafish. Meanwhile, an inhibitor of MAP4K4/6/7 can mediate YAP activity in mice, drive gene expression of proneural transcription factors to activate RMCs, and facilitate retinal regeneration. **C, D** In retinal gliosis-related blindness, diabetes-driven YAP-overactivation in RMCs induces its activation and the dysregulation of glutamate recycling, resulting in aggravated retinal fibrosis, photoreceptor dysfunction, and tractional retinal detachment. ACs, amacrine cells; Akt, v-akt murine thymoma viral oncogene homologue; BCs, bipolar cells; Crb, crumbs homolog; CTGF, connective tissue growth factor; Cyr61, encoding cysteine-rich angiogenic inducer 61; GCs, ganglion cells; GCL, ganglion cell layer; HCs, horizontal cells; INL, inner nuclear layer; IPL, inner plexiform layer; MAP4K, mitogen-activated protein kinase kinase kinase kinases; ONL, outer nuclear layer; OPL, outer plexiform layer; PI3K, phosphoinositide 3-kinase; PPP1CA, protein phosphatase 1 catalytic subunit alpha; PRP-Exos, platelet-rich plasma-derived exosomes; RMCs, retinal Müller cells; TGF-β, transforming growth factor β; TRULI, The Rockefeller University Lats Inhibitor.

During retinal development, RMCs and retinal neurons originate from progenitors in a temporally stereotyped manner [86]. Ganglion cells, amacrine cells, horizontal cells, and cone photoreceptors are produced by early progenitors around birth, followed by late progenitors-produced rod photoreceptors, bipolar cells, and RMCs after birth [87]. TRULI (The Rockefeller University Lats Inhibitor), *via* the small-molecule inhibition of Lats kinases, activates YAP and remarkably amplifies the proliferative capacity of RMCs [88] (Fig. 5A). The polarity proteins Crb1 and Crb2 form the Crumbs complex which is located in adherent junctions between retinal progenitor cells or photoreceptors and RMCs. Chx10 (a retinal homeobox gene, which is expressed in retinal progenitors and adult RMCs)-Cre; Crb1; Crb2 knockout mice exhibit retinal degeneration and vascular defects resulting from the mislocalization, proliferation, and apoptosis of late-born cells, including RMCs. In these mice, YAP, which acts on the proliferation of the progenitor cells in the postnatal retina, is downregulated, leading to a reduction in its downstream target *CTGF* and *Cyr61* (encoding cysteine-rich angiogenic inducer 61) [89]. The deficiency of YAP by Chx10-Cre makes a parallel phenotype with normal retinal development but leads to abnormal visual responses and extensive late‐onset retinal degeneration of both rod and cone photoreceptors [90]. Likewise, aged YAP^+/−^ mice, created using PKG-Cre and YAP ^flox/+^ mice, exhibit impaired cone visual function due to retinal dysplasia and late-onset cone degeneration caused by the deficiency of YAP and the epidermal growth factor receptor (EGFR). However, this YAP deficiency can be compensated for by TAZ during postnatal development [91].

#### Retinal Müller Cell Reprogramming

The dysfunction of RMCs results in photoreceptor cell death, ultimately leading to neurodegenerative retinal diseases (Fig. 5B). Nonetheless, RMCs regenerate photoreceptors through reprogramming in zebrafish and *Xenopus*, a process in which RMCs proliferate and transfer to a self-renewing state of progenitors followed by migration to and differentiation in the lesion site [92, 93]. In zebrafish, YAP in RMCs participates in the regeneration of the damaged retina by supporting RMC proliferation, progenitor cell amplification, and differentiation in YAP/lin28a/ascl1a, two *bona fide* Müller glia-reprogramming factors, and the YAP/EGFR axis, proteins well-known for mitogenic effects [94]. However, in mammals, the proliferative response to injury is largely finite [95]. Emerging evidence has shown that there is a YAP-related mechanism in mammalian/rodent RMCs to promote their reprogramming and proliferation. Mechanistically, inhibition of mitogen-activated protein MAP kinase kinase kinase kinases (MAP4K) 4/6/7 has been shown to mediate YAP activation and the YAP/EGFR pathway in RMCs by promoting RMC reactivation, proliferation, de-differentiation, and reprogramming into retinal neurons [96–98].

#### Retina-related Diseases

In blinding conditions that involve retinal gliosis, such as proliferative vitreoretinopathy (PVR) and diabetic retinopathy (DR), YAP is remarkably upregulated [99] (Fig. 5C, D). Understanding the mechanisms underlying YAP may encourage the development of novel therapies for retinal diseases. Upon ocular trauma and retinal detachment, PVR may culminate in modification of the substrate elastic modulus [100]. In immortalized RMC cell lines, nuclear YAP/TAZ cooperates with maximum CTGF expression in a bimodal relationship with the elastic modulus [99, 101]. DR is a common but specific microvascular impairment in diabetes, which induces pathological neovascularization and ECM production and eventually results in photoreceptor dysfunction and tractional retinal detachment [102, 103]. Zhang *et al.* [104] reported a feedback-loop process between stiffness and YAP in which diabetic retinal fibrosis-induced greater ECM stiffness causes an increase of YAP expression in the RMCs while YAP activation escalates ECM production. During this, the TGF-β1/PI3K/v-akt murine thymoma viral oncogene homologue (Akt)/YAP pathway gradually aggravates diabetes-induced retinal fibrosis. Similarly, platelet-rich plasma-derived exosomes isolated from the plasma of diabetic rats activate YAP through PI3K/Akt, thereby enhancing the expression of CTGF and augmenting both proliferation and fibrogenic RMC activity *in vitro* [105]. In the facet of metabolism in the retina, glutamine is inferred to play a role in the metabolic dysregulation of DR *via* glutamate recycling. Under hyperglycemic conditions, RMCs are activated and proliferate *via* the protein phosphatase 1 catalytic subunit alpha (PPP1CA)/YAP/glutamine synthetase/glutamine/mTORC1 pathway during DR [106].

### YAP in Bergmann Glia (BG)

**Fig. 6:**
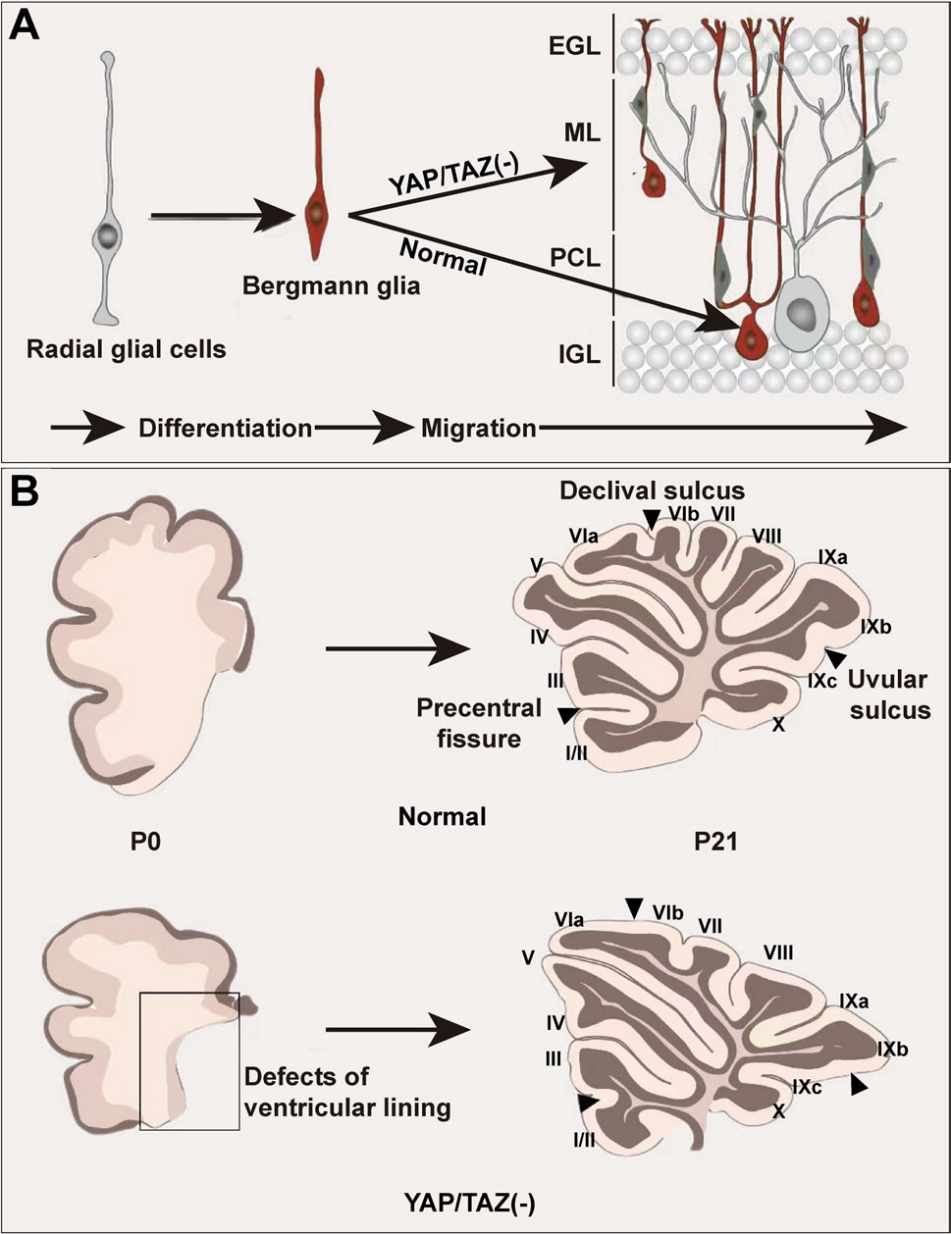
The roles of YAP in Bergmann glia. In the early development of the cerebellum, YAP in BG helps pinpoint the migration of BG (**A**), which is beneficial for the subsequent migration of granule neuron progenitors and cerebellar foliation. However, dysfunction of YAP in BG induces defects in migration, as their cell bodies are mislocalized to the molecular layer rather than in the PC layer and cause incorrect migration to the molecular layer (**B**). The cerebellum in YAP/TAZ^Nestin^ CKO mice shows defects in the ventricular lining at postnatal day 0 and defects of the precentral fissure between lobules II and III at postnatal day 21. EGL, external granular layer; IGL, internal granular layer; ML, molecular layer; P, postnatal day; PCL, Purkinje cell layer.

The BG is composed of unipolar protoplasmic astrocytes, located on the cell bodies of Purkinje cells (PCs) to extend radial or BG fibers enwrapping synapses on PC dendrites in the cerebellar cortex [107]. During development, RGCs lose their apical processes, become BG, and translocate to the PC layer. The BG fibers function to guide neuronal migration with granule cells [108]. Notably, a strong expression of YAP in BG is readily seen during early developmental stages and in the adult cerebellum (Fig. 6). At postnatal day 0, BG of Nestin-Cre; YAP; TAZ CKO mice exhibit defects in migration, as their cell bodies are mislocalized to the molecular layer rather than the PC layer. Notably, the ventricular lining in Nestin-Cre; YAP; TAZ CKO mice is missing at this phase. Besides, PCs, BG, and granule cells can form ‘anchoring centers’, which are important for cerebellar foliation, thereby establishing the basis of the characteristic cerebellar fissures. At postnatal day 10, severe foliation defects are accompanied by significantly fewer BG in lobules II and III, which is most likely due to the decreased length of these lobules, but not the number or density. As a scaffold, long radial BG promotes migration in maturing granule cells from the external granular layer to the inner granule layer. However, wild-type and YAP/TAZ CKO mice are comparable at postnatal day 21 and later stages, suggesting a YAP/TAZ requirement only for normal progression to establish the BG layer at the PC layer [76].

### YAP in Astrocytes

Astrocytes are the most abundant brain cell type, which plays myriad crucial roles including basic structural and metabolic support for neurons, regulation of synaptic transmission, vasomodulation, maintenance of the blood-brain barrier, and long-term potentiation [109].

#### Neocortical Astrogliogenesis

**Fig. 7:**
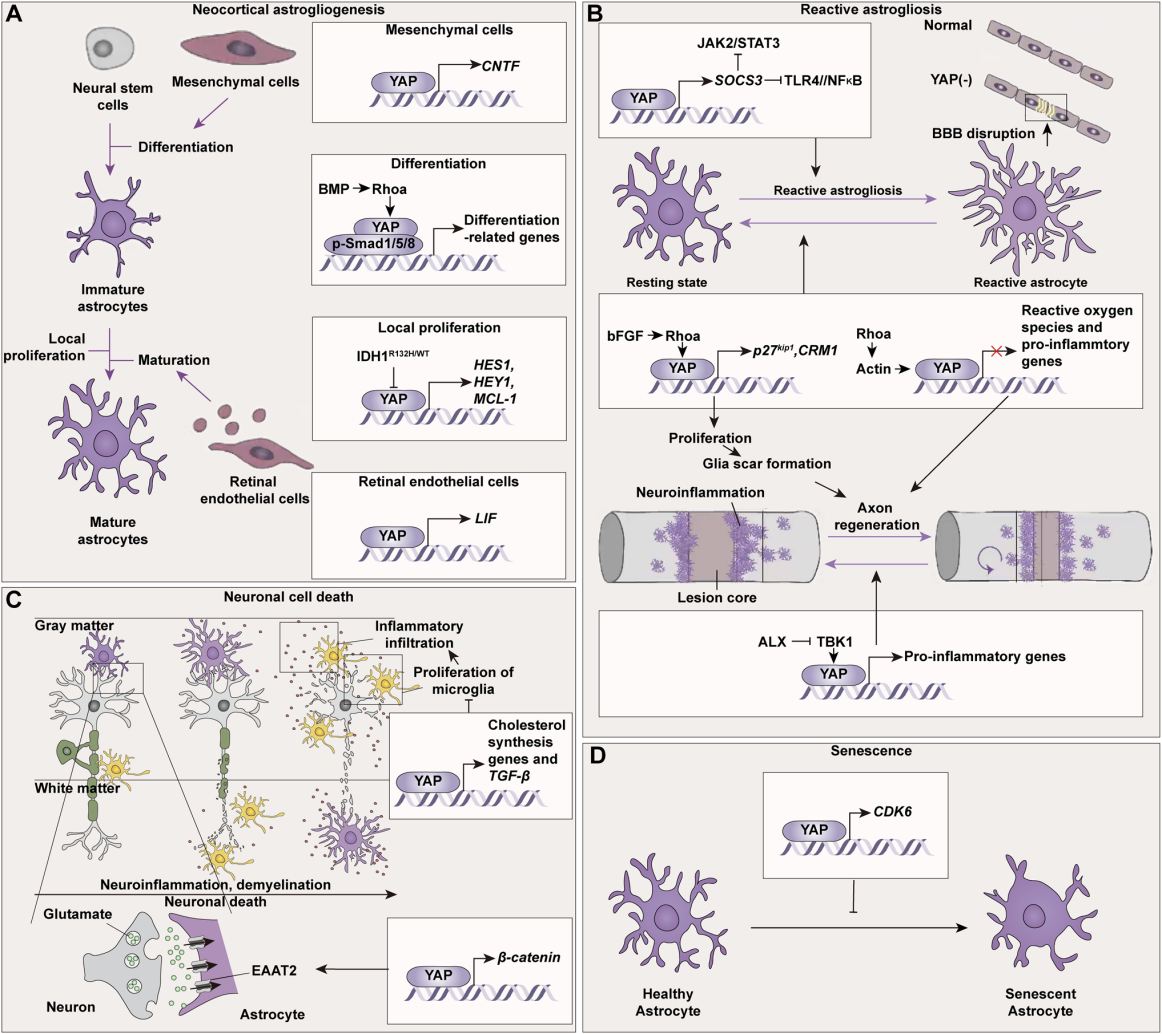
The roles of YAP in astrocytes. **A** YAP from astrocytes and other adjacent cells promotes the differentiation, local proliferation, and maturation of developing astrocytes (purple arrow). The YAP/CNTF pathway in mesenchymal cells and BMP/Rhoa-induced astrocytic YAP promotes neocortical astrocytic differentiation. The IDH1^R132H/WT^ mutation downregulates YAP to trigger an anti-proliferative role. YAP in retinal endothelial cells enhances the secretion of LIF to contribute to the maturation of developing astrocytes. **B** CNS injuries trigger changes in YAP and induce reactive astrogliosis where YAP acts as a double-edged sword (purple arrow). YAP suppresses reactive astrogliosis through SOCS1/3 associated with blood-brain barrier dysfunctions. YAP is also involved in scar formation after SCI through the bFGF/RhoA/YAP/CRM1/p27^Kip1^ pathway, which is beneficial to the regeneration of axons and functional recovery. Conversely, inhibited nuclear YAP in astrocytes attenuates a reactive astrogliosis-mediated neuroinflammatory response after traumatic SCI by the TBK1 inhibitor amlexanox (purple arrow). **C, D** Moreover, YAP is involved in delaying senescence, activating microglia, and neuroinflammation during demyelinating diseases with enhanced transcription of CDK6, TGF-β, β-catenin, and cholesterol synthesis genes, which may be critical features in Alzheimer’s disease and other degenerative diseases (purple arrow). ALX, amlexanox; BBB, blood-brain barrier; CDK6, cyclin-dependent kinase 6; CNS, central nervous system; CNTF, ciliary neurotrophic factor; EAAT2, excitatory amino-acid transporters 2; JAK2, Janus kinase 2; LIF, leukemia inhibitory factor; NF_K_B, nuclear factor-k-gene binding; SOCS3, suppressor of cytokine signaling; STAT3, signal transducer and activator of transcription 3; TBK1, TANK-binding kinase 1; TGF-β, transforming growth factor β; TLR4, Toll-like receptor 4.

Our previous work showed that astrocytic YAP is expressed in both the developing neocortex and hippocampus, yet surprisingly appears to be only required for the neocortex [24, 110, 111]. This is probably due to their diverse sources. Astrocytes are derived from the neural stem cells that reside in the ventricular zone. Across rodents, cortico-cerebral astrogliogenesis mainly occurs sequentially following proliferation and neurogenesis. This astrogliogenesis contains two simultaneous processes: astrocytic differentiation and the local proliferation of astrocytes [112] (Fig. 7A). Using astrocytic YAP CKO mice including YAP^Nestin^ CKO and YAP^GFAP^ CKO mice, we found that YAP promotes the above two processes to increase neocortical astrogliogenesis through BMP (a member of the TGF-β superfamily of signaling ligands)/neogenin/YAP/SMAD1 pathway [24, 111]. Moreover, the gain-of-function of YAP by introducing YAP 5SA *in utero* induces astrocytic differentiation by increasing the expression of ciliary neurotrophic factor (CNTF, an important inducer of reactive gliosis) in a non-cell-autonomous manner [25]. In a separate study, Wei *et al.* [113] successfully generated heterozygous isocitrate dehydrogenase 1 R132H mutation by a highly efficient “C-to-T” single-base editing approach in human astroglial cells. After mutation, YAP and its downstream Notch pathway are downregulated to trigger an anti-proliferative role.

#### YAP in Retinal and Optic Nerve Head Astrocytes

The retina and optic nerve are crucial components of the CNS. YAP deficiency in endothelial cells impairs retinal astrocytic maturation by reducing the secretion of leukemia inhibitory factor (LIF). Further investigation in oxygen-induced retinopathy once again demonstrated and raised the point that endothelial YAP can protect the astrocyte network and even prevent pathological retinal vascularization [114, 115]. In the optic nerve head (ONH), a region where the retinal ganglion cell axons exit the eye without myelin wrapping, astrocytes surround these unmyelinated axons in the form of honeycomb-like networks [116, 117]. Wan *et al.* [118] reported that loss of the mechanosensitive ion channel Piezo1 decreases astrocytic YAP nuclear localization and downregulates the cell cycle-associated proteins cyclin D1 and c-Myc, thereby inducing the cell-cycle arrest of ONH astrocytes.

#### Reactive Astrogliosis

Under stress or pathological insults, astrocytes are activated and transform into a special type of immune neuroglia (so-called reactive astrogliosis). The reactive astrocytes release substantial pro- or anti-inflammatory cytokines, chemokines, and neutrophils to provoke tissue damage or repair (Fig. 7B) [119]. YAP suppresses the reactive astrogliosis by regulating the expression of suppressor of cytokine signaling 1/3 (SOCS1/3) in response to cytokine stimulation (e.g., interferon-β and CNTF). The absence of YAP lessens the signal transducer and activator of the transcription 3 (STAT3) signaling pathway, a pathway recognized as the central regulator of reactive astrogliosis. This may negatively control the microglia activation and neuroinflammation associated with blood-brain barrier dysfunctions [21].

In the spinal cord injury (SCI) mice model, Stern *et al.* [120] showed that RhoA activates YAP signaling by driving actin in astrocytes to constrain their reactivity. In the intracerebral hemorrhage model, the Cx43 mimetic peptide Gap19 suppresses both the abnormal protein expression of Cx43 and the excessive opening of Cx43 hemichannels without Cx43 transcription in astrocytes, facilitates YAP-dependent SOCS1 and SOCS3 expression, and then inhibits two inflammatory signaling pathways, the toll-like receptor 4/NFκB and Janus kinase 2/STAT3 pathways, to protect cell survival against reactive astrocytes after intracerebral hemorrhage [121]. Conversely, hemoglobin exposure downregulates Cx43 but increases nuclear YAP, where it transcriptionally enhances proliferation and mesenchymal features, an early event in reactive astrogliosis [122]. Following a traumatic SCI, amlexanox (a specific inhibitor of TANK-binding kinase 1) influences innate immune NFκB pathways and phosphorylates interferon regulatory factor 3 *via* YAP. The reduced nuclear translocation of YAP subsequently restrains the reactive astrogliosis-mediated neuroinflammatory response and loss of motor neurons, demonstrating the contribution of YAP to reactive astrogliosis [123]. These bilateral characters of YAP due to the diverse stages of neuroinflammation or distinct models of physiological or pathological processes need to be further elucidated.

#### Glial Scar

Scar formation after CNS injury is essential for the recovery of damaged tissue and constraints on damage, whereas it is also a barrier to regeneration [124]. Following SCI, activated astrocytes surround the outer layer of the fibrous scar and compose the primary glial component of the scar [50]. A pioneering study of LATS1 in SCI implied a prospective role of YAP [125]. In line with this, we found that YAP activation is gradually induced after SCI. Basic fibroblast growth factor (bFGF), a potent mitogenic and chemotactic factor, that is upregulated in SCI, promotes the proliferation of astrocytes, the formation of the glial scar, the regeneration of axons, and functional recovery after SCI through the bFGF/RhoA/YAP/CRM1/p27^Kip1^ (a cyclin-dependent kinase enzyme inhibitor) axis [126].

#### Neuronal Cell Death

In both the *in vivo* middle cerebral artery occlusion model and *in vitro* oxygen-glucose deprivation/reperfusion model, astrocytic YAP prevents neuronal death, deterioration of the neurological deficit, and the eventual reduction of cerebral infarction through the STAT3 pathway [127]. Moreover, astrocytic YAP signaling is also required for the expression of excitatory amino-acid transporters 2 (EAAT2), a glutamate transporter mainly expressed in astrocytes, and is vital for maintaining glutamate homeostasis [128]. Dysregulation of YAP suppresses EAAT2 *via* β-catenin, ultimately triggering neuronal death and cognitive deterioration [129] (Fig. 7C).

#### Demyelinating Disease

Experimental autoimmune encephalomyelitis (EAE) is the most widespread experimental model of multiple sclerosis (MS), the most common demyelinating disease of the CNS in young adults. Astrocytes are activated within demyelinating lesions and participate in key processes of MS and EAE [130, 131]. Our data revealed that astrocytic YAP prevents demyelination and neuroinflammation in the retina and optic nerve of EAE through upregulating TGF-β signaling (regulating inflammatory immune cells and myelination). Specifically, deficient YAP enhances microglial activation and inflammatory infiltration [132]. In support of this, the YAP/3-hydroxy-3-methylglutaryl-CoA synthase 1 pathway for cholesterol synthesis in astrocytes also prevents the neuroinflammation and demyelination in the spinal cord during EAE [133].

#### Senescence

From the aspect of senescence, recent findings in an aged SCI model reveal a significant increase in the levels of phosphorylated YAP (p-YAP) and the p-YAP/YAP ratio, which is correlated with severe motor dysfunction. This condition is mitigated by knockout of the P75 neurotrophic receptor. [26]. Even in mouse models of Alzheimer's disease (AD), YAP acts as an inhibitor to partially postpone the senescence of astrocytes through cyclin-dependent kinase 6 (CDK6) signaling to improve cognitive function [16] (Fig. 7D).

### YAP in Microglia

**Fig. 8:**
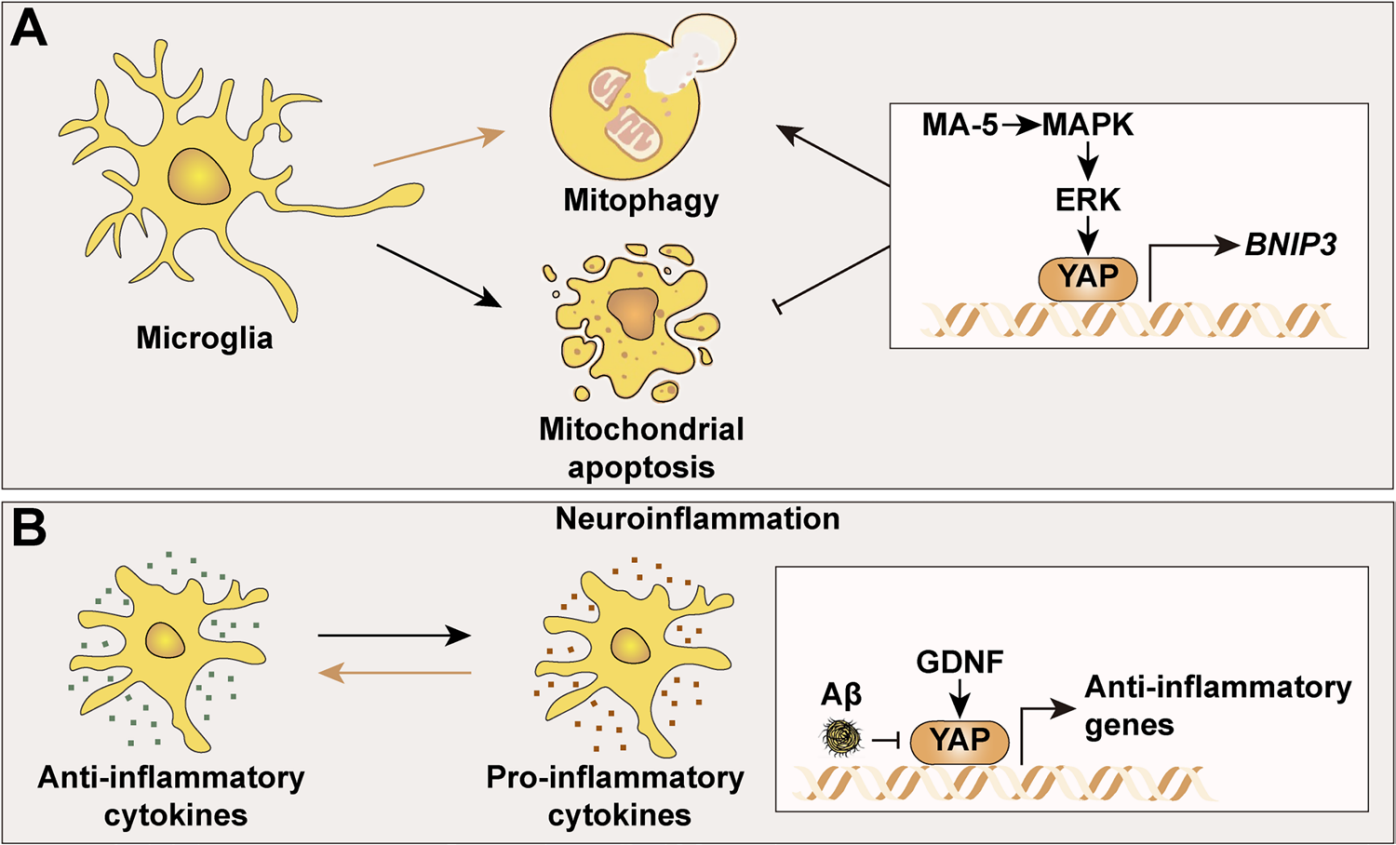
The roles of YAP in microglia. **A** Schematic showing that MA-5 facilitates mitophagy and inhibits mitochondrial apoptosis through the MAPK/ERK/YAP/BNIP3 pathway. **B** GDNF upregulates YAP in microglia against the Aβ-induced downregulation of YAP and the pro-inflammatory state, which may be of much help in relieving neuroinflammation (yellow arrow). Aβ, amyloid-β; ERK, extracellular signal-related kinases; GDNF, glial cell line-derived neurotrophic factor; MA-5, mitochonic acid 5; MAPK, mitogen-activated protein kinases.

Microglia are the primary immune cells located in the CNS, best known as the brain’s resident macrophages. As ‘protectors’ of the CNS, microglia shift their phenotype according to the microenvironment, allowing them to sustain their role in brain homeostasis (Fig. 8) [109]. Mitochondrial acid 5 (MA-5) orchestrates BCL2/adenovirus E1B 19-kDa protein-interacting protein 3 (BNIP3, activating mitophagy) *via* the mitogen-activated protein kinases (MAPK)/extracellular signal-related kinases (ERK)/YAP pathway. These consequences boost microglial BV-2 cell line mitophagy but hinder TNFα-induced mitochondrial apoptosis, which may confront inflammatory response-mediated microglial death [134]. Kindlin 3 (K3), which controls integrin activation, is a protein expressed in hematopoietic cells [135]. K3 deficiency or disruption of K3-integrin and K3-paxillin/actin binding in myeloid cells containing microglia and macrophages diminish YAP translocation to the nucleus during membrane-to-cortex attachment [136].

A decade of intense research pointed out that neuroinflammation may be a critical mechanism for the progression of AD. As the majority of players involved in the inflammatory process, microglia can be activated by amyloid-β (Aβ) and release a series of pro-inflammatory mediators [137]. Compared to normal cells, Aβ-induced microglia have a reduced expression of YAP. Glial cell line-derived neurotrophic factor (GDNF, having neurotrophic and antiapoptotic actions on the nervous system and inhibition of the expression of pro-inflammatory mediators) can downregulate YAP expression to strengthen the effect against the Aβ-induced inflammatory response in microglia [138]. Traumatic brain injury (TBI), which is characterized by secondary paroxysmal sympathetic hyperactivity, is an illness that affects nearly half of the world’s population in the course of their lifespan [139]. Zhu *et al.* [140] discovered that the formation of neutrophil extracellular traps in the paraventricular nucleus might be related to sympathetic hyperactivity after TBI, on account of the activation of microglia and the subsequent increased IL-1β *via* the decreased MST1 and YAP.

### YAP in Oligodendrocytes (OLs)

**Fig. 9:**
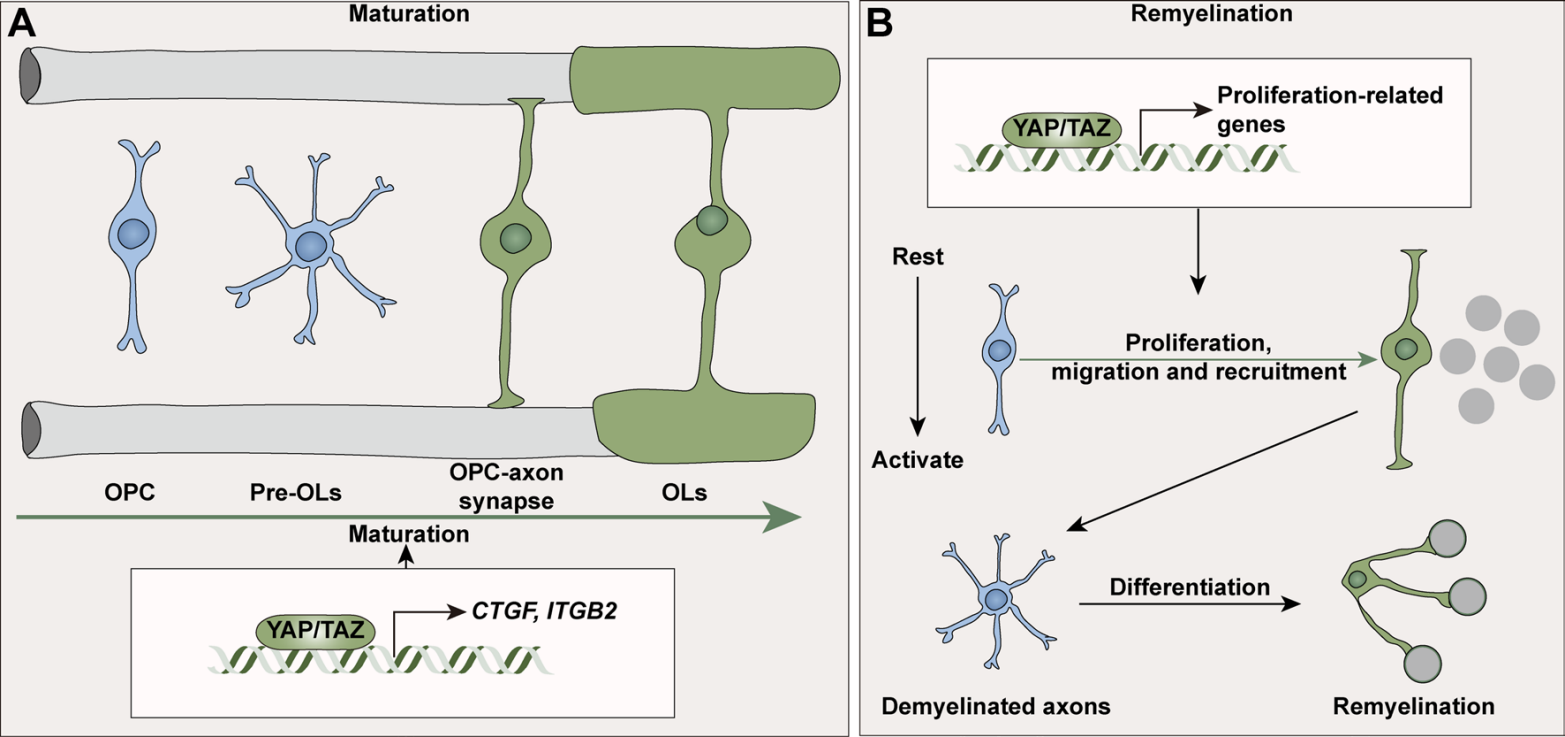
The roles of YAP in oligodendrocytes. YAP/TAZ promotes the maturation of oligodendrocytes *via* the upregulation of CTGF and ITGB2. In demyelination/remyelination models, YAP/TAZ functions in the proliferation of OLs, which is helpful for recovery at the early stage (green arrow). CTGF, connective tissue growth factor; ITGB2, integrin subunit beta 2; OPC, oligodendrocyte precursor cell; pre-OLs, pre-myelinating oligodendrocytes.

OLs are myelinating cells of the CNS [141], while the role of YAP in OLs largely differs from that in SCs (Fig. 9). Co-culture of the DRG model with YAP shRNA OLs or mechanical stress on purified oligodendrocyte precursor cells (OPCs) presents a specific role of mechanical cues in extending processes to contact neuronal axons *via* YAP nuclear translocation. Nevertheless, DRGs are the sensory transducers in the PNS, which may lead to separate effects of axons in the CNS on OLs.

In mouse optic nerve, YAP is expressed between P2 and P21 but less at P21, when synaptic connections form, thereby reducing mechanical factors. Different from *in vitro* experiments, overexpressing YAP by PLP-tTA; YAP-STOP-tetO mice triggers excessive YAP accumulation in nucleus, which in turn upregulates the mechanical downstream signals CTGF and integrin subunit beta 2. This overexpression affects the morphogenesis of these cells and ultimately impairs the maturation of white matter [142]. Interestingly, Hong *et al.* [143] detected YAP/TAZ in both the optic nerve and corpus callosum during P6 to P90, where OLs are abundantly distributed. They found that YAP is low from P6 to P10 (beginning of myelination) but higher at P15 to P90 in the optic nerve while remaining stable in the corpus callosum. In line with this, YAP participates in the mechanical stiffness-related effect of myelinating OLs but does not differentiate OPCs [144]. Regarding the function of YAP, PLP1-Cre^ER^; YAP^f/f^; TAZ^f/f^, or oligodendrocyte transcription factor (OLIG) 2-Cre; YAP^f/f^; TAZ^f/f^ mice do not differ in myelination and brain development from control mice, implying the lack of YAP/TAZ does not change OL maturation and myelination. After application of the demyelination/remyelination model, these two kinds of knockout mice show slower proliferation of OLs, which may fail to act in the early stage of recovery, leading to graver demyelination [143].

## Conclusions and Future Perspectives

**Fig. 10:**
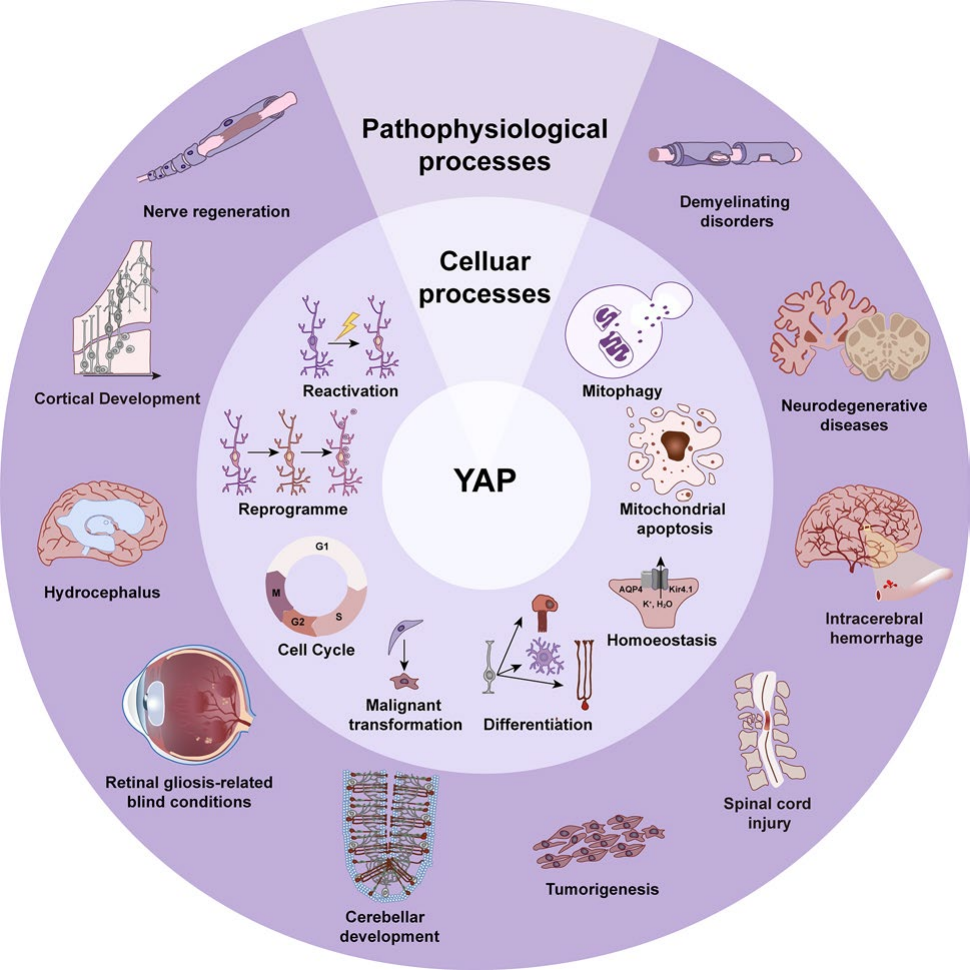
Graphic Abstract. YAP in glia-related cellular and pathophysiological processes. Schematic of the main cellular and pathophysiological events coincident with YAP expression.

In summary, YAP functions in different temporal or spatial changes in diverse glial cell types through various target proteins and genes, and in physiological or pathological processes. The role of YAP has been studied throughout the years to clarify its vital role in glial proliferation, differentiation, maturation, senescence, homeostasis, nerve injury, and neurodegenerative diseases (Fig. 10). However, many mysteries remain. The effect of YAP mainly focuses on SCs, RMCs, and astrocytes, but the mechanisms underlying the divergent expression in multifarious models remain unclear as well as their roles in different brain regions. Moreover, how they act on or are affected by neighboring cells, especially other glial cells, is still elusive.

As to applications in clinical treatment, YAP has sparked much interest in mechanotransducers. Cells are intrinsically responsive to physical stimuli, including substantial tension forces (mediated by cytoskeletal elements) and intermittent local compression forces (mediated by ECM and neighboring cells) [145]. To foster peripheral nerve repair, an innovative silk-inspired fiber scaffold (RGD@ASFFs) that enhances the bionic microenvironment, has been developed. The cornerstone of this approach lies in the combination of mechanical cues and cell adhesion signals, ultimately leading to enhanced myelination of SCs by the nuclear translocation of YAP [146]. Low-intensity extracorporeal shock wave therapy, a mechanical treatment that has been applied to many clinical pathological conditions, has been found to YAP/TAZ-dependently promote SC proliferation [147]. However, the combination of YAP and mechanical cues poses a substantial challenge to maintaining proper levels of YAP. Furthermore, as a bilateral actor in neurological disorders, YAP needs to be precisely tuned to a safe zone. Overall, given the vast potential of YAP in glia, there is room for optimism: the role and mechanism of YAP in glial cells and their related diseases will gradually be elucidated and fundamentally effective new clinical approaches will be discovered.
